# Supplementary figures and images for: Recent updates on metabolite composition and medicinal benefits of mangosteen plant
Source: PeerJ. 2019 Jan 31;7:e6324. doi: 10.7717/peerj.6324 (PMC6368837; doi:10.7717/peerj.6324)

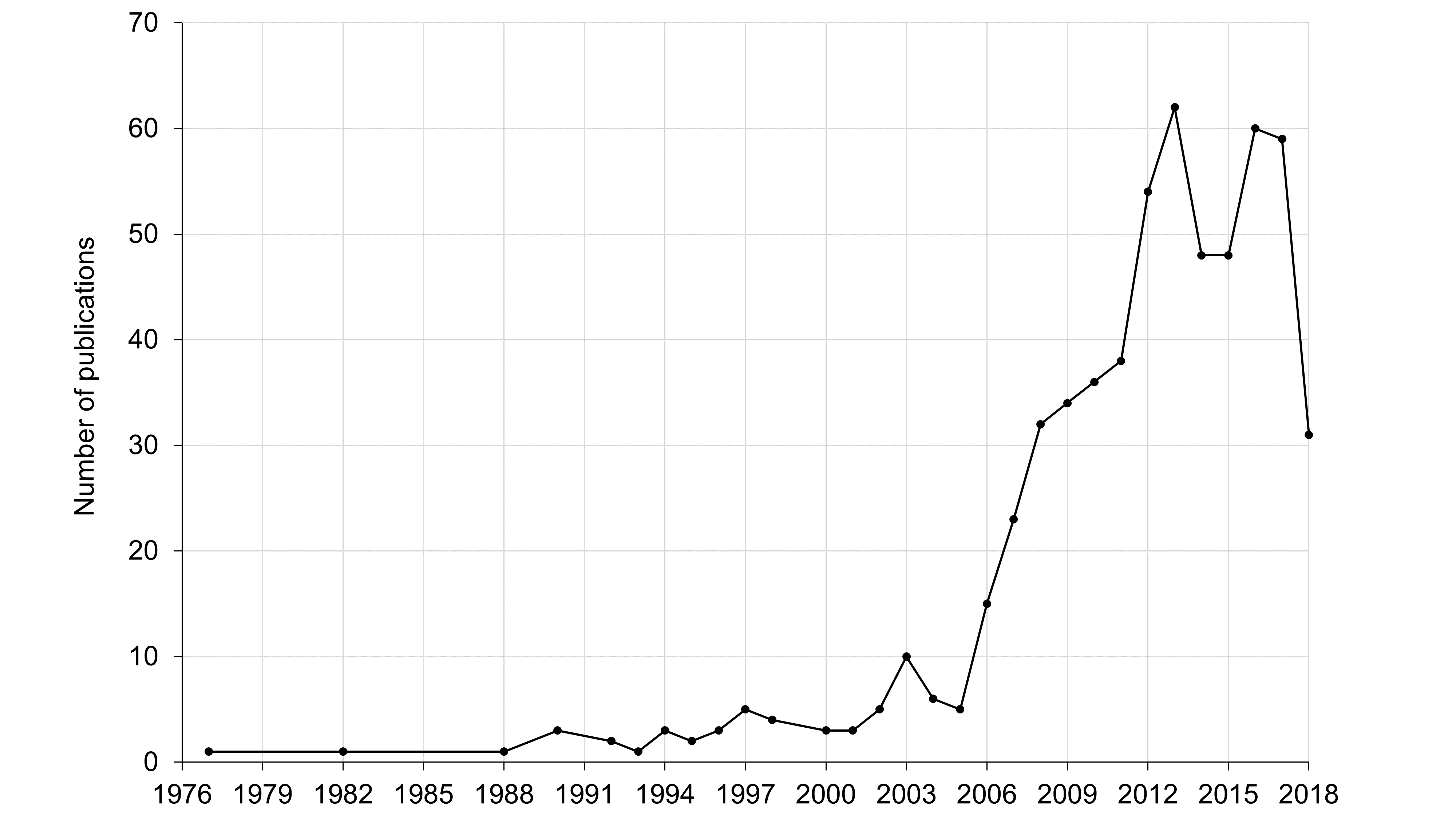

Supplement: Supplemental Information 1 — Statistics were obtained from SCOPUS database on July 2018 by searching “mangosteen AND Garcinia mangostana” in the “Article title, Abstract and Keywords” search field. Please refer to Supplementary File 1 for the raw data. [file peerj-07-6324-s001.png]

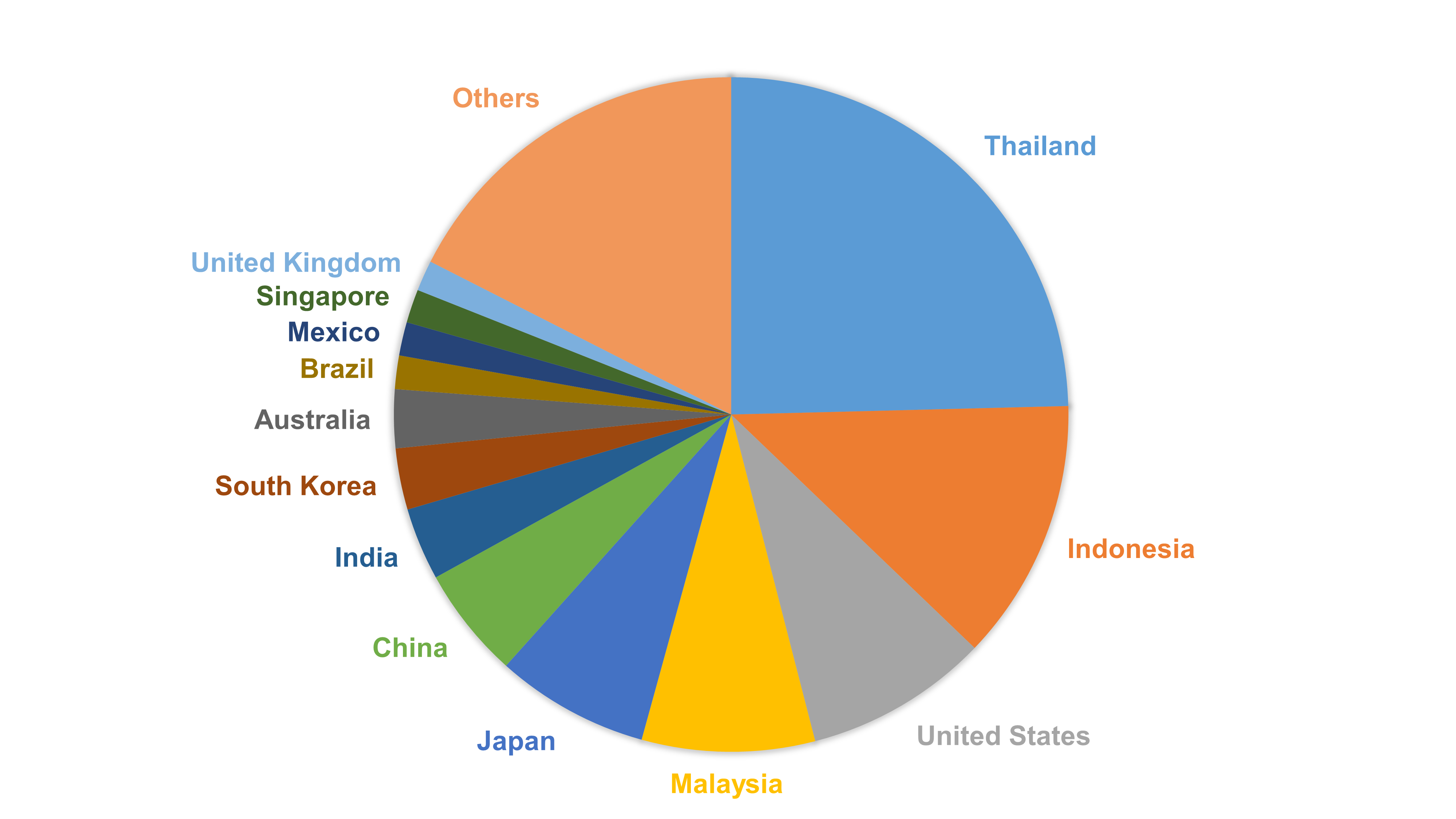

Supplement: Supplemental Information 2 — Statistics were obtained from SCOPUS database on July 2018 by searching “mangosteen AND Garcinia mangostana” in the “Article title, Abstract and Keywords” search field. [file peerj-07-6324-s002.png]
